# Association of Sequential Organ Failure Assessment (SOFA) components with mortality

**DOI:** 10.1111/aas.14067

**Published:** 2022-04-10

**Authors:** Anssi Pölkki, Pirkka T. Pekkarinen, Jukka Takala, Tuomas Selander, Matti Reinikainen

**Affiliations:** ^1^ Department of Anaesthesiology and Intensive Care Kuopio University Hospital Kuopio Finland; ^2^ University of Eastern Finland Kuopio Finland; ^3^ Division of Intensive Care Medicine Department of Anaesthesiology, Intensive Care and Pain Medicine Helsinki University Hospital University of Helsinki Helsinki Finland; ^4^ Department of Intensive Care Medicine University Hospital Bern (Inselspital) University of Bern Bern Switzerland; ^5^ Science Service Center Kuopio University Hospital Kuopio Finland

**Keywords:** Multiorgan Failure, SOFA, SOFA score, SOFA score components, SOFA score weights, Surrogate endpoint

## Abstract

**Background:**

Sequential Organ Failure Assessment (SOFA) is a practical method to describe and quantify the presence and severity of organ system dysfunctions and failures. Some proposals suggest that SOFA could be employed as an endpoint in trials. To justify this, all SOFA component scores should reflect organ dysfunctions of comparable severity. We aimed to investigate whether the associations of different SOFA components with in‐hospital mortality are comparable.

**Methods:**

We performed a study based on nationwide register data on adult patients admitted to 26 Finnish intensive care units (ICUs) during 2012−2015. We determined the SOFA score as the maximum score in the first 24 hours after ICU admission. We defined organ failure (OF) as an organ‐specific SOFA score of three or higher. We evaluated the association of different SOFA component scores with mortality.

**Results:**

Our study population comprised 63,756 ICU patients. Overall hospital mortality was 10.7%. In‐hospital mortality was 22.5% for patients with respiratory failure, 34.8% for those with coagulation failure, 40.1% for those with hepatic failure, 14.9% for those with cardiovascular failure, 26.9% for those with neurologic failure and 34.6% for the patients with renal failure. Among patients with comparable total SOFA scores, the risk of death was lower in patients with cardiovascular OF compared with patients with other OFs.

**Conclusions:**

All SOFA components are associated with mortality, but their weights are not comparable. High scores of other organ systems mean a higher risk of death than high cardiovascular scores. The scoring of cardiovascular dysfunction needs to be updated.

**Editorial Comment**

In this large study from the Finnish ICU registry, evidence is provided to show poor performance of the cardiovascular component of the SOFA score. The authors suggest that a revision of this sub‐score relative weight might improve the predictive value of the overall score for mortality.

## 1 INTRODUCTION

The Sequential Organ Failure Assessment (SOFA), at first named Sepsis‐related Organ Failure Assessment, was introduced by The Working Group on Sepsis‐Related Problems in 1996.^1^ The SOFA score describes and quantifies the severity of dysfunction or failure of six essential organ systems (Table S1). Primarily, the SOFA score was not meant for outcome prediction. Multiple studies have shown, however, that it can rather well predict mortality in groups of critically ill patients.^1^, ^2^, ^3^, ^4^, ^5^, ^6^, ^7^, ^8^, ^9^, ^10^, ^11^, ^12^, ^13^ This has notably widened the employment of the SOFA score beyond its original purpose.

In randomised controlled trials, the gold standard has been to use all‐cause mortality as an endpoint. However, interventional trials often fail to detect any difference between study arms in mortality.^14^ Therefore, there is growing interest to use surrogate endpoints, for example SOFA scores.^15^, ^16^, ^17^ Regulatory authorities, including the European Medicines Agency, can under certain limitations approve the use of surrogate endpoints instead of mortality as primary endpoints.^18^

The change in the SOFA score during critical illness has been proposed to reflect the benefit or harm of the intervention of interest. The SOFA score, which is a scalar variable, is presumably more sensitive in detecting the effects of an intervention than mortality, a binary variable. However, the total SOFA score cannot be an unbiased trial endpoint unless all its components have comparable weights as measures of organ dysfunction severity. Moreover, some organ failures (OFs) are more likely to occur concurrently.^3^ It is unclear whether different combinations of OFs affect the predictive value of total SOFA score.

The aim of this study was to investigate whether different SOFA score components, recorded during the first 24 h of intensive care, carry comparable weights in terms of their association with mortality. In other words, do patients with comparable total SOFA scores have comparable probabilities to perish regardless of which OFs they suffer from? We evaluated how combinations of different organ system failures are associated with mortality. Furthermore, we assessed the association of increasing SOFA scores with mortality across different admission groups. Mortality at hospital discharge was the primary endpoint. Mortality at ICU discharge and mortality within 12 months were secondary endpoints.

## 2 METHODS

### 2.1 Study design and participants

The study protocol was approved by the Research Ethics Committee of the Northern Savo Hospital District Data (225/13.02.00/2016), and research authorisation was obtained from the National Institute for Health and Welfare (THL/1585/5.05.00/2015). Due to the retrospective nature of the study, the Research Ethics Committee waived the written informed consent in line with Finnish act of personal data.

We performed a retrospective cohort study of data collected prospectively in the Finnish ICU quality register, the Finnish Intensive Care Consortium (FICC) database. The FICC is a national programme for benchmarking intensive care in Finland.^19^ FICC covers all 26 general ICUs of central and university‐level hospitals in Finland.

We included all adult patients admitted to Finnish ICUs between January 1, 2012 and December 31, 2015. For patients with multiple ICU treatment periods during the same hospitalisation, we included only the first ICU admission. In line with the 1998 paper by the working group that created the SOFA system,^2^ we defined OF as an organ‐specific SOFA score of three or higher. OF could appear isolated or as part of multiorgan failure.

We performed subgroup analyses to observe whether the findings were consistent, regardless of the admission type—medical, elective surgery and emergency surgery.

### 2.2 Extracted variables

We extracted following variables from the FICC database: the most severe values of SOFA score components within the first 24 h after admission to the ICU and the outcome variables: vital status at ICU discharge, at hospital discharge and 12 months after ICU admission. Moreover, we gathered baseline data on Acute Physiology, Age, Chronic Health Evaluation (APACHE) II,^20^ The Simplified Acute Physiology Score (SAPS) II,^21^ age and sex. We also retrieved data on length of stay in the ICU and length of stay in hospital.

### 2.3 Data handling and statistical methods

In the neurologic component, the SOFA score is based on the Glasgow Coma Score (GCS). For anaesthetised or sedated patients, the GCS recorded to the FICC registry is the last reliable GCS preceding sedation, in line with the SAPS II score.^21^

The hepatic SOFA score is based on the plasma bilirubin concentration. Bilirubin is normally measured when there is a clinical reason to suspect hepatic problems. Therefore, we consider normality of bilirubin concentrations as likely in patients for whom the data on bilirubin were missing. In these patients, we assumed the hepatic SOFA score to be 0. We made no assumption of normality for other SOFA components in cases of missing data. Therefore, we excluded patients with missing SOFA data concerning all other components except for the hepatic component. In addition, we excluded patients with missing mortality data.

We compared the characteristics of survivors and non‐survivors at hospital discharge employing the Mann−Whitney U‐test for continuous data and chi square test for categorical data. Using age‐adjusted multivariable logistic regression, we evaluated the association between SOFA score components and mortality. All components as well as age were included in the analysis simultaneously. P‐value of less than 0.05 was considered as statistically significant in all tests. We calculated standardized occurrence ratio (SOR) for each set of at least two, three, or four concurrently occurring failing organ systems. SOR is a tool to evaluate whether particular OFs occur concurrently more frequently than anticipated by merely observing the frequencies of OFs. SOR was calculated as $N\left(o\right)÷\left[N\times p\left(a\right)\times p\left(b\right)\right]$, where $N\left(o\right)$ is the number of patients with OF of *a* and *b*, N is the total number of admissions and $p\left(a\right)$ and $p\left(b\right)$ are the proportions of patients with failure of organ systems a andb, respectively. In the same way, we calculated the SOR for patients with three and four concurrent OFs. SOR >1 signals that the odds of concurrent occurrence of these particular failing organ systems are increased. Bonferroni correction was used for multiple comparisons regarding the SOR analysis.

We used IBM SPSS Statistics, Version 22 (IBM Corp., Amonk, NY, USA) and R statistical software version 4.0.4 for the statistical analyses.

## 3 RESULTS

### 3.1 Study population

**FIGURE 1:**
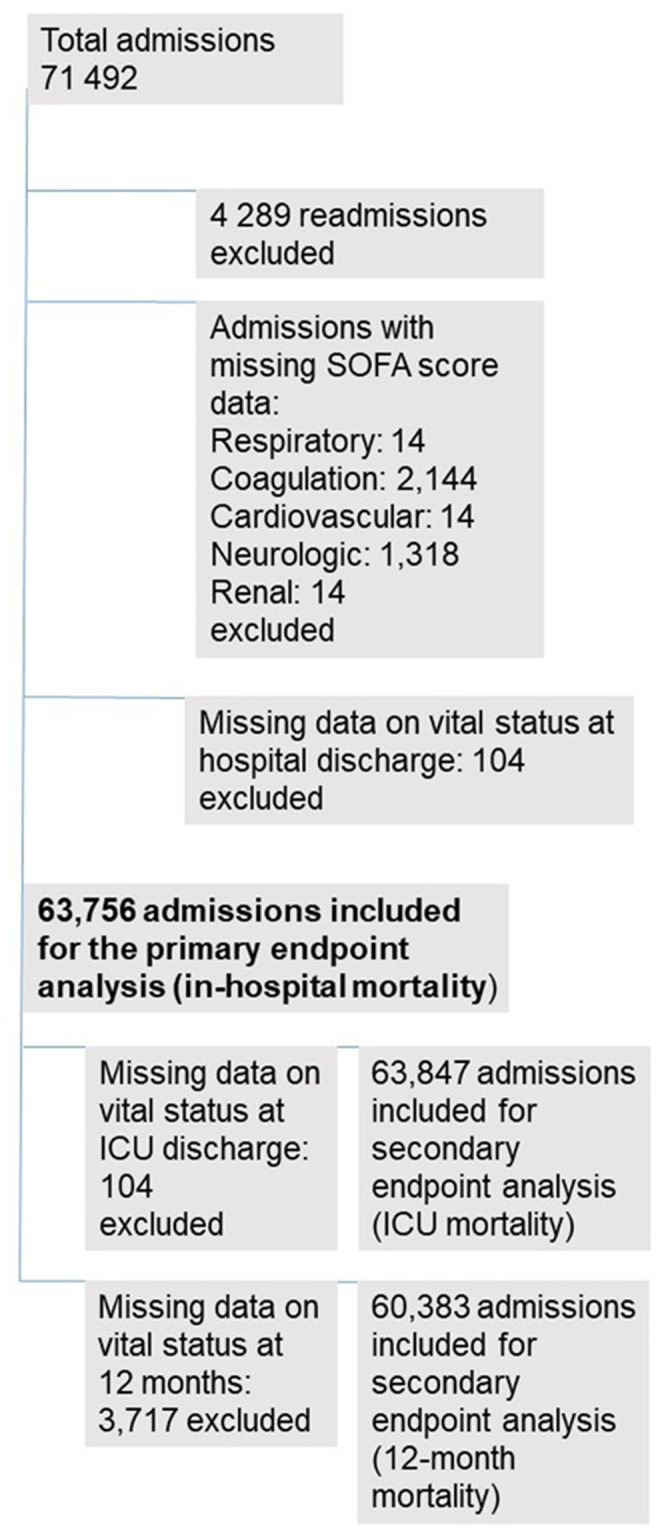
Flowchart

There were totally 71,492 ICU admissions during the study period. We excluded 4289 (6%) readmissions. Data were missing most commonly for the hepatic component, for 26,435 (39.3%) admissions. For other components, data were missing for few admissions: 14 (0%) in respiratory, 2144 (3.2%) in coagulation, 14 (0%) in cardiovascular, 1318 (2%) in neurologic and 14 (0%) in the renal component. We excluded 104 (0.2%) cases with missing data on vital status at hospital discharge. The final study population included 63,756 patients (Figure 1). For ICU and 12‐month mortality calculations, we excluded 16 (0%) cases with missing data on vital status at ICU discharge and 3,717 (5.5%) cases with missing data on vital status at 12 months, respectively.

**FIGURE 2:**
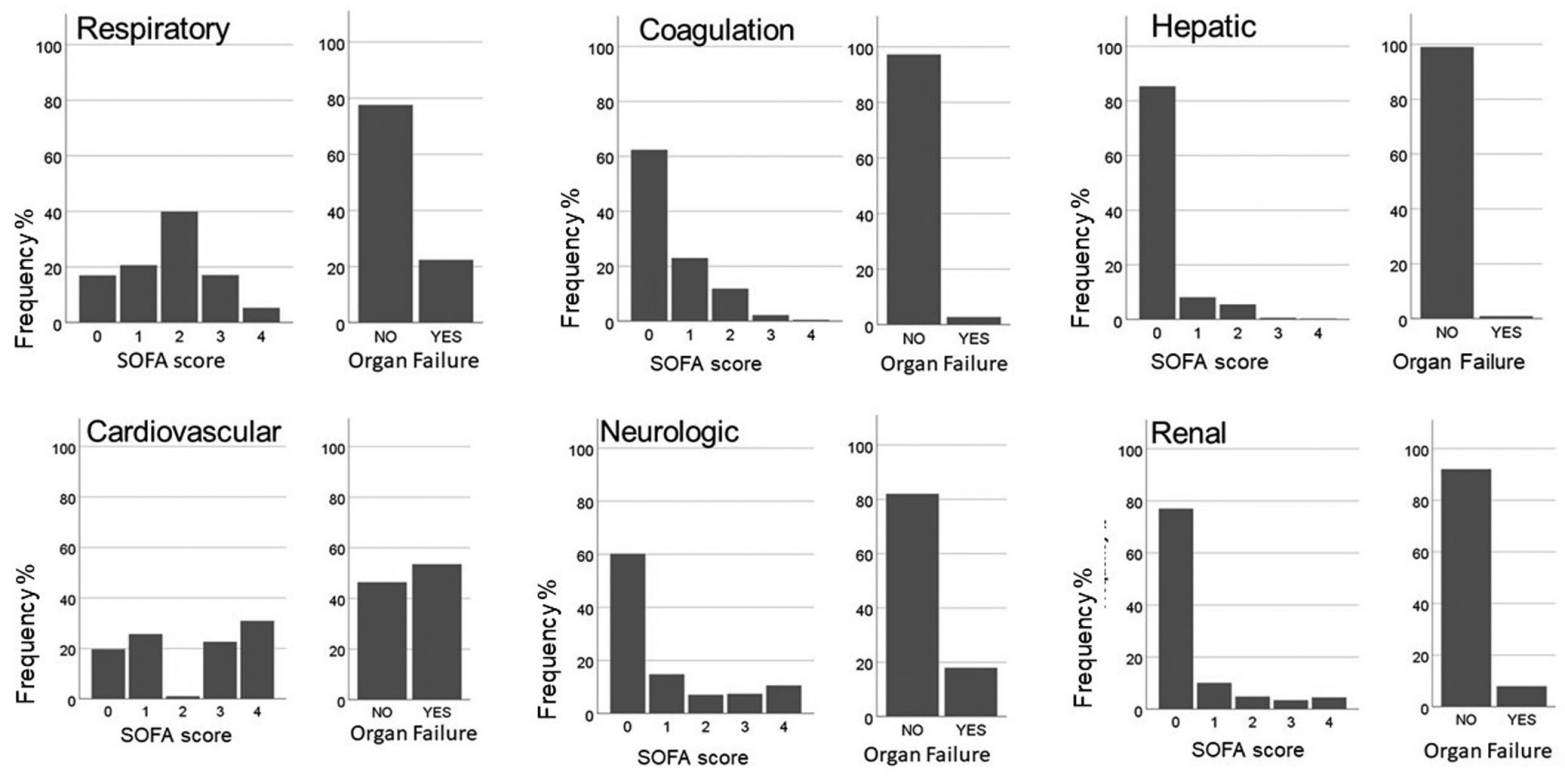
The distribution of SOFA component scores and frequency of organ failures

The median age of the patients was 64 years (inter‐quartile range 52–73), and the majority (63.7%) were male. During the ICU stay, 66.9% of the patients needed mechanical ventilation and 6.1% renal replacement therapy. Baseline data are presented in Table 1. The median score in the respiratory component was 2 and in the cardiovascular component 3. In the cardiovascular component, the scores were almost equally distributed among the patients except for score 2, which was documented for only 678 (1.1%) patients. In all other components (coagulation, hepatic, neurological and renal), the median score was 0, with the score 1 being second most common (Figure 2). Of OFs, defined as an organ‐specific SOFA score ≥3, the most common OF was cardiovascular failure, in 53.6% of patients. The second most common OF was respiratory failure, in 22.5% of patients.

**TABLE 1:** Demographics, baseline characteristics and lengths of stay in ICU and hospital

	Overall (n=63,756)	Survivors (n=56,905)	Non‐survivors (n=6,851)	p‐value
Age, median (IQR)	64 (52–73)	63 (51–73)	69 (61–77)	<0.001
Female, n (%)	23 121 (36.3%)	20 642 (36.3%)	2 479 (36.2%)	0.87
SOFA	6 (4–8)	6 (3–8)	10 (7–12)	<0.001
SAPS II	31 (23–44)	29 (22–40)	56 (44–69)	<0.001
APACHE II	18 (13–24)	17 (12–22)	29 (24–35)	<0.001
Metastatic cancer	1 550 (2.4%)	1 295 (2.3%)	255 (3.7%)	<0.001
Haematologic malignancy	886 (1.4%)	650 (1.1%)	236 (3.6%)	<0.001
AIDS	71 (0.1%)	60 (0.1%)	11 (0.2%)	0.02
Admission type				<0.001
Medical	34 987 (55.2%)	29 651 (52.5%)	5 336 (78.1%)	
Elective surgery	17 034 (26.8%)	16 774 (29.6%)	260 (3.8%)	
Emergency surgery	11 401 (17.9%)	10 165 (17.9%)	1236 (18.1%)	
Diagnostic category				<0.001
Cardiovascular surgery	15 130 (23.7%)	14 619 (25.7%)	517 (7.5%)	
Neurologic	10 753 (16.9%)	9 756 (17.1%)	997 (14.6%)	
Cardiovascular insufficiency	8 967 (14.1%)	6 651 (11.7%)	2 316 (33.8%)	
Metabolic or renal	6 862 (10.8%)	5 961 (10.5%)	901 (13.2%)	
Respiratory insufficiency	5 989 (9.4%)	4 987 (8.8%)	1 002 (14.6%)	
Gastrointestinal surgery	4 886 (7.7%)	4 288 (7.5%)	598 (8.7%)	
Trauma	4 316 (6.8%)	4 043 (7.1%)	273 (4.0%)	
Other postoperative cause	2 769 (4.3%)	2 664 (4.7%)	105 (1.5%)	
Intoxication	2 666 (4.2%)	2 615 (4.6%)	51 (0.7%)	
Miscellaneous	1 397 (2.2%)	1 307 (2.3%)	90 (1.3%)	
LOS ICU (days), median (IQR),	1.4 (0.9–3.1)	1.3 (0.9–2.9)	2.1 (0.9–5.1)	<0.001
LOS Hospital (days), median (IQR)	8 (5–14)	8 (5–14)	5 (2–13)	<0.001

Note

Data are presented as numbers with percentages or as medians (inter‐quartile ranges). Characteristics of hospital survivors and non‐survivors were compared with the Mann−Whitney U‐test for continuous data and Chi‐squared test for categorical data.

Abbreviations: AIDS, acquired immune deficiency syndrome; APACHE, acute physiology and health evaluation; ICU, intensive care unit; LOS, length of stay; QR, Inter‐quartile range; SAPS, Simplified Acute Physiology Score; SD, standard deviationSOFA, Sequential Organ Failure Assessment.

### 3.2 ICU, hospital and 12‐month mortality

**FIGURE 3:**
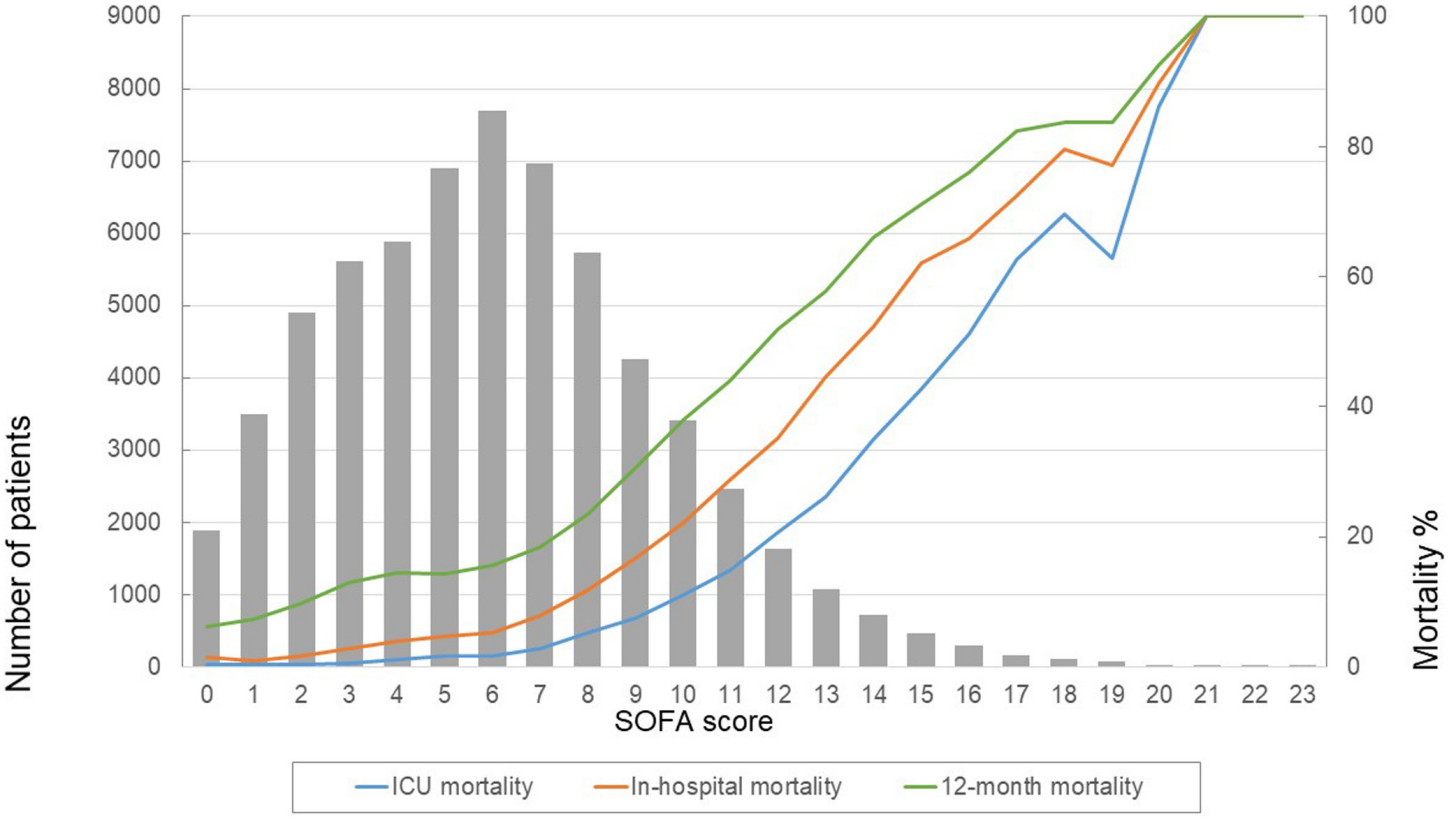
The number of patients and mortality according to first‐day total SOFA score. ICU mortality (blue line), in‐hospital mortality (red line) and 12‐month mortality (green line) increased with increasing total SOFA score. The bars present the number of patients within each total SOFA score group

Overall, 6,851 (10.7%) patients died in hospital. The first day total SOFA score was strongly associated with mortality (Figure 3). Mortality was 5.3% at ICU discharge and 21.6% in 12 months. Mortality increased with increasing SOFA scores (Figure 3). In‐hospital mortality was 15.0% in those patients with LOS at the ICU more than 48 h. There were 642 (1%) patients with a SOFA score over 15. In these patients, ICU mortality was 60%, hospital mortality was 72%, and 12‐month mortality was 80%.

**FIGURE 4:**
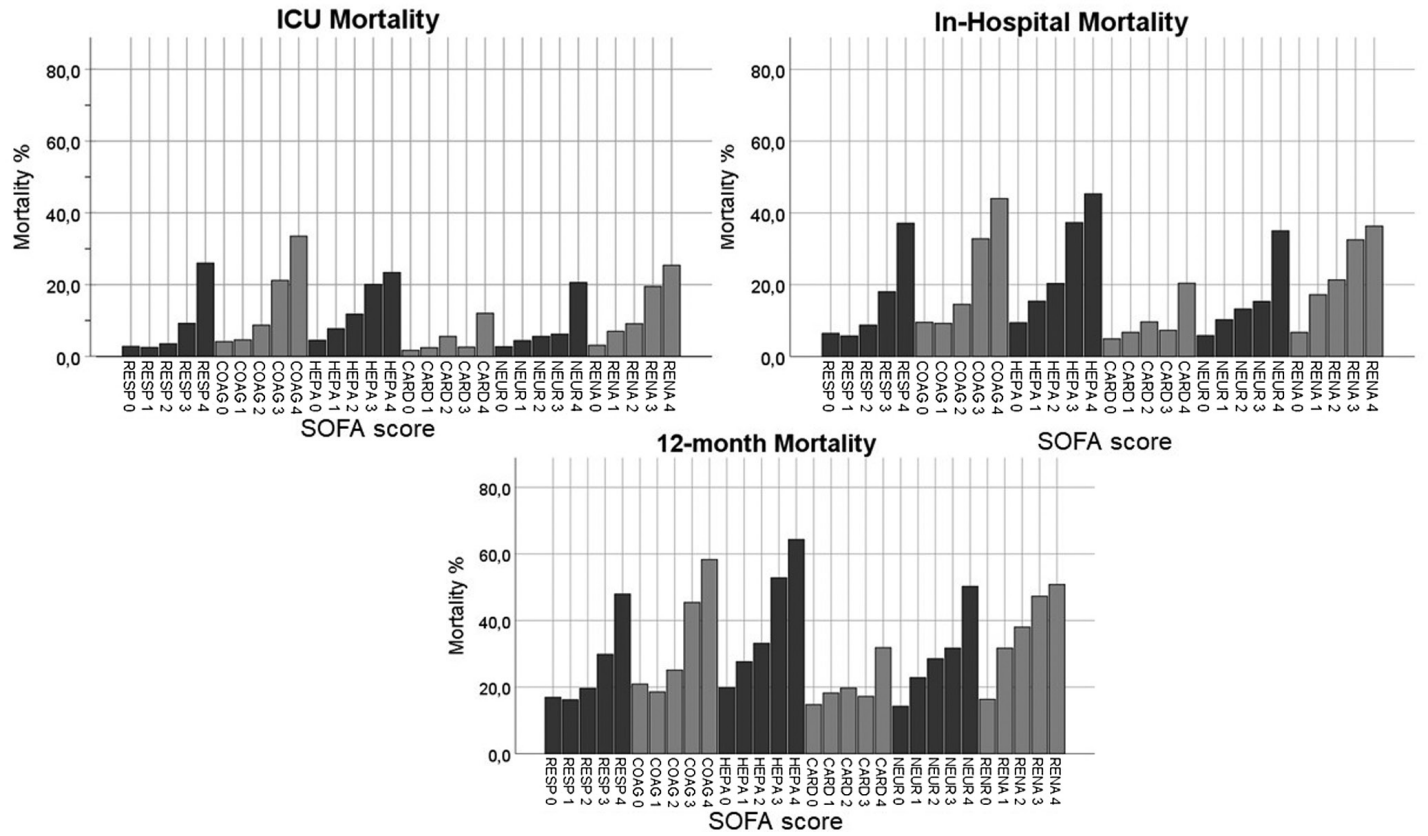
ICU mortality, in‐hospital mortality and 12‐month mortality according to SOFA component scores. ICU mortality, in‐hospital mortality and 12‐month mortality are presented in separate panels. The bars present the mortality in each SOFA score category recorded in the first 24 hours after ICU admission

**FIGURE 5:**
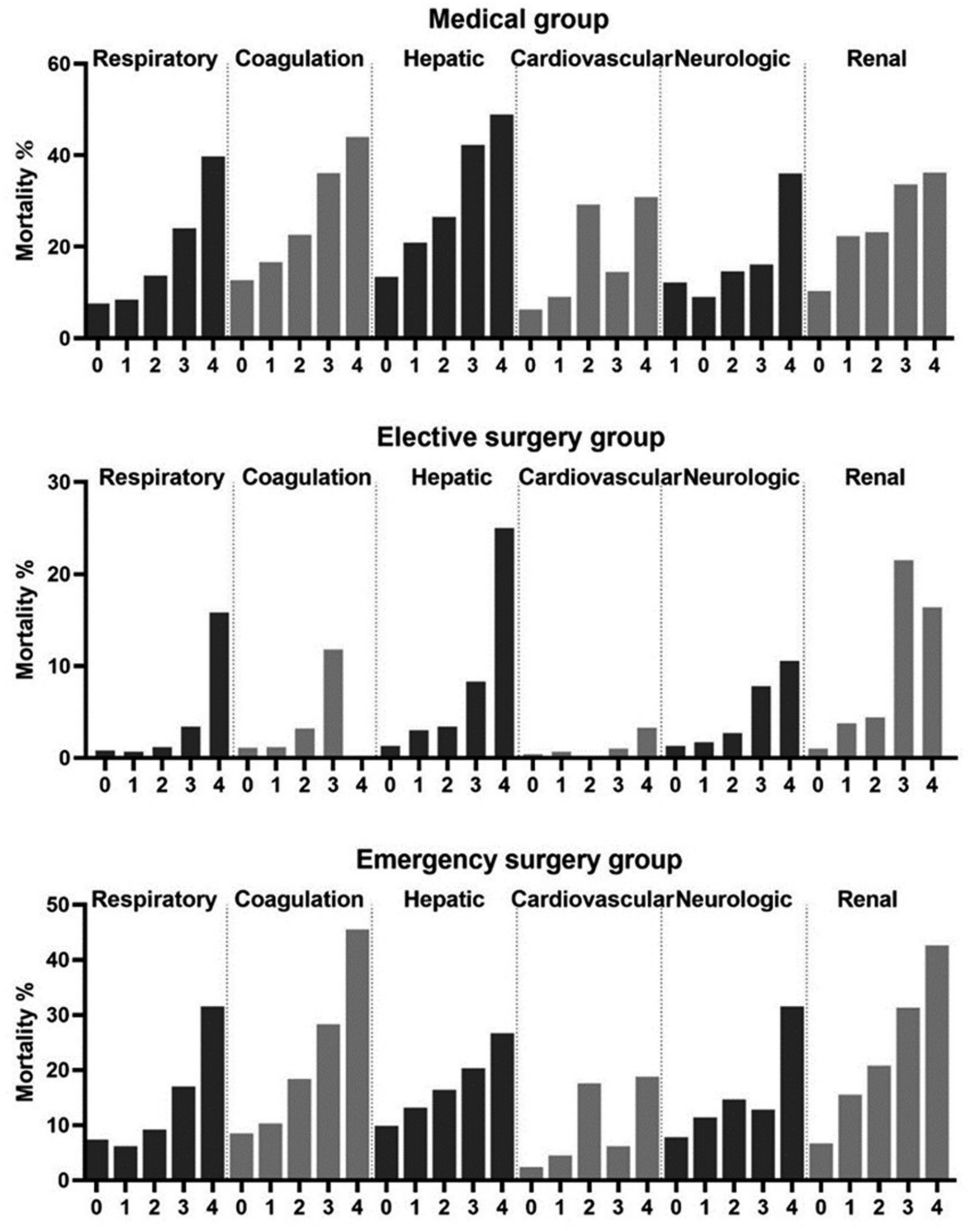
Hospital mortality according to SOFA component scores in different admission categories. The bars present the in‐hospital mortality according to SOFA component scores in different admission categories (medical, elective surgical and emergency surgical). Hospital mortality increased with increasing SOFA component scores

Mortality mostly increased consistently with increasing SOFA component points (Figure 4). The cardiovascular component, however, was an exception. In this component, a clear increase in mortality occurred only in the group with the score 4. For the respiratory and coagulation components, mortality was similar for the scores of 0 and 1 points but increased consistently with increasing points thereafter. This pattern appeared rather similar regardless of whether the vital status was observed at ICU or hospital discharge or 12 months after ICU admission (Figures 4 and 5).

**FIGURE 6:**
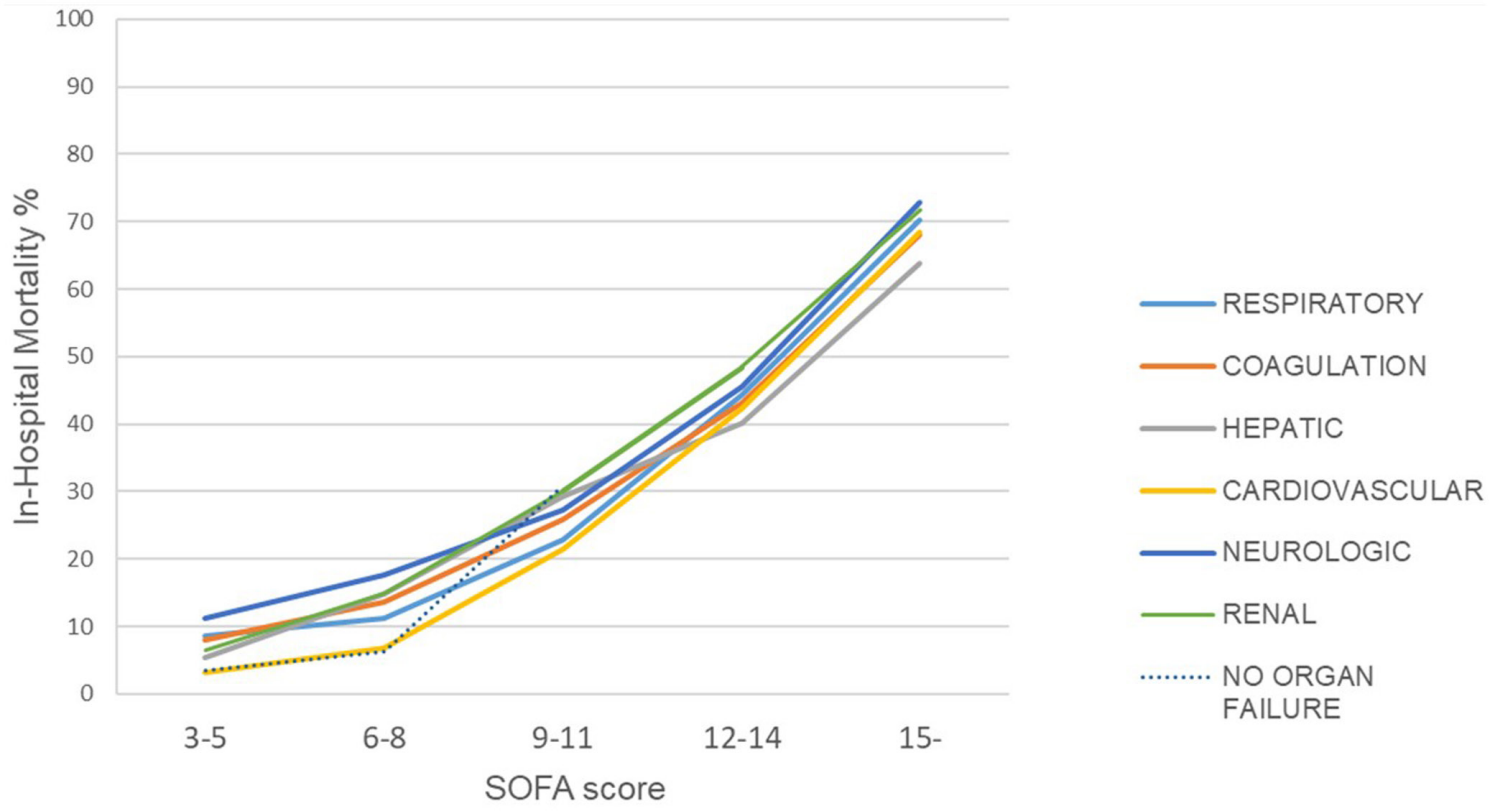
Mortality in patients with different organ failures according to total SOFA score. The lines represent in‐hospital mortality in patients with respiratory (light blue), coagulation (orange), hepatic (grey), cardiovascular (yellow), neurologic (purple) and renal (green) failure. The organ failure was determined as organ‐specific SOFA score 3 or 4. Mortality in patients without any first‐day organ failure is shown with black dashed line

Mortality in patients with OFs (organ‐specific SOFA score 3 or 4) increased with increasing total SOFA scores. However, within groups of patients with comparable total SOFA scores, mortality was lower in patients with cardiovascular OF compared with patients with other OFs in patients with a total SOFA score lower than 12. In fact, mortality in patients with cardiovascular OF did not exceed the mortality in patients with no first‐day OF at all in patients with a total SOFA score lower than 9 (Figure 6).

Respiratory failure was observed for 16 277 (22.8%) patients, coagulation failure for 1 932 (2.7%), hepatic failure for 704 (1.0%), cardiovascular failure for 37 672 (52.7%), neurologic failure for 12 714 (17.8%), and renal failure for 5 958 (8.3%) patients (Figure 2). Hospital mortality was 22.5% for patients with respiratory failure, 34.8% for those with coagulation failure, 40.1% for those with hepatic failure, 14.9% for those with cardiovascular failure, 26.9% for those with neurologic failure and 34.6% for the patients with renal failure. Concerning patients with LOS more than 48 h, the in‐hospital mortality was 20.4% for patients with respiratory failure, 30.1% for those with coagulation failure, 36.2% for those with hepatic failure, 17.1% for those with cardiovascular failure, 22.3% for those with neurologic failure, and 24.6% for the patients with renal failure.

The results of age‐adjusted multivariable logistic regression analysis are presented in Table 2. The odds of in‐hospital death were highest for patients with neurologic failure, whereas the odds of death were lowest in patients with cardiovascular failure. Especially for 12‐month outcome, cardiovascular OF had little influence on the risk of death.

**TABLE 2:** The association of failures of different organ systems and age with ICU, hospital and 12‐month mortality

	ICU mortality			Hospital mortality			12‐month mortality		
	OR	95% CI		OR	95% CI		OR	95% CI	
Respiratory OF	2.92	2.70	3.16	2.41	2.27	2.56	1.71	1.63	1.79
Coagulation OF	4.18	3.63	4.82	4.04	3.57	4.57	3.24	2.891	3.64
Hepatic OF	2.27	1.78	2.89	4.24	3.47	5.17	4.27	3.53	5.17
Cardiovascular OF	2.15	1.95	2.36	1.57	1.47	1.67	1.05	1.01	1.10
Neurologic OF	4.63	4.28	5.01	5.00	4.71	5.30	4.13	3.93	4.34
Renal OF	5.99	5.48	6.55	4.93	4.58	5.32	3.81	3.56	4.07
Age (for each year)	1.01	1.01	1.02	1.03	1.03	1.04	1.04	1.04	1.04
Female sex	1.16	1.07	1.26	1.02	0.96	1.08	0.95	0.91	0.99

Note

Abbreviations: CI, confidence intervalOF, organ failure; OR, odds ratio.

### 3.3 Combinations of organ system failures and mortality

**FIGURE 7:**
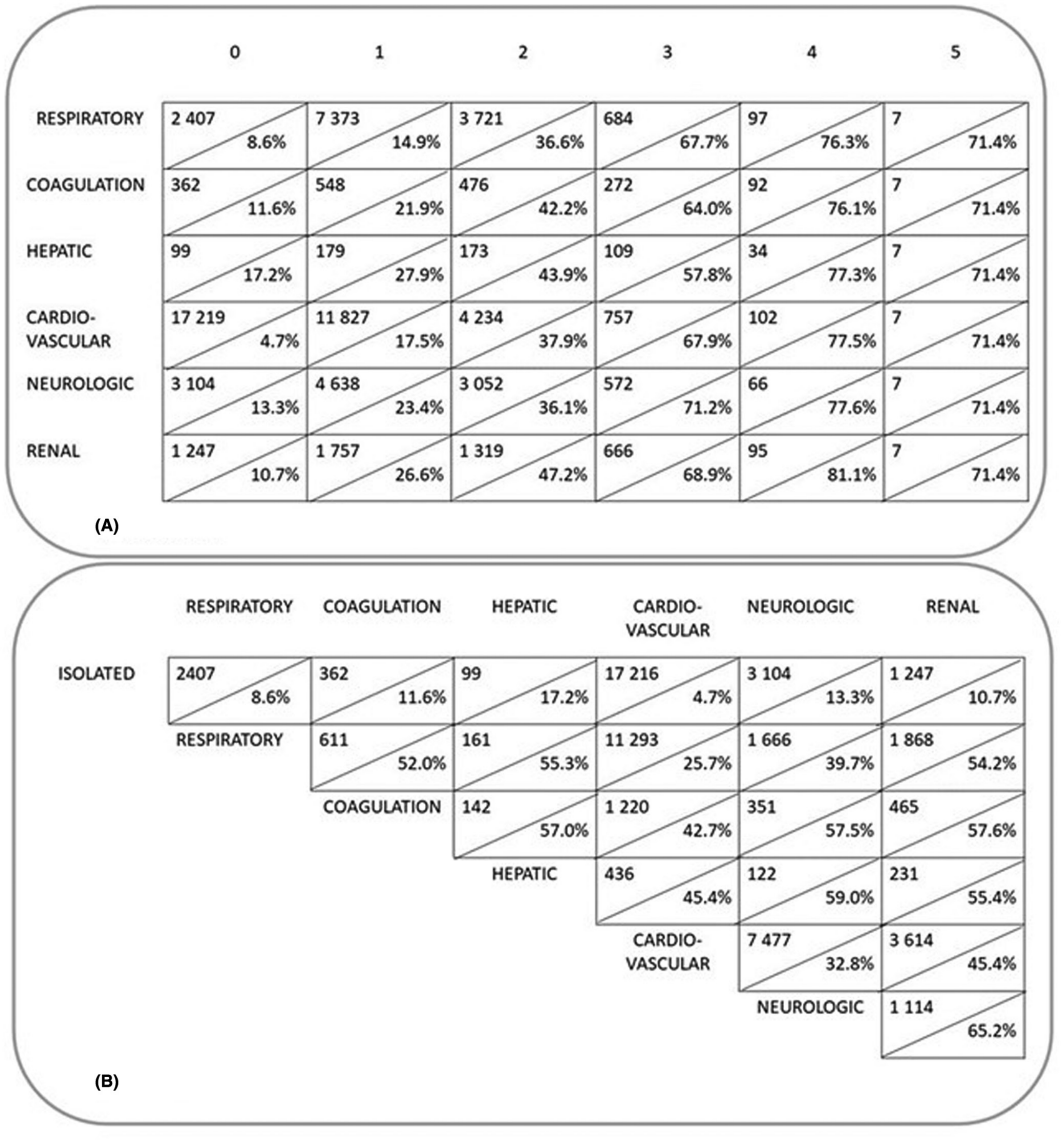
Mortality according to the number of failing organ systems and mortality in groups with at least two simultaneous organ failures. In panel a, each organ failure is represented by a line. In each box, the number above the diagonal line presents the number of patients with the column title‐presented number of additional failing organ systems in addition to the organ failure of that line. The percentage below the diagonal line presents the in‐hospital mortality of these patients. Panel b presents the number of patients with a combination of at least two organ failures. The percentage below the diagonal line shows the in‐hospital mortality in patients with that particular combination

Mortality increased with increasing numbers of concurrent OFs (Figure 7). Of all patients, 47.4% had at least two, 12.7% had at least three and 2.0% had at least four concurrent OFs. In‐hospital mortalities in these groups were 35.8%, 54.1% and 71.8%, respectively. SOR was >1 in 48 (94.1%) out of all 51 OF combinations (Table S2), suggesting that OFs are likely to occur concurrently. In‐hospital mortality ranged between 25.7%–65.2% in patients with two, 41.4%–82.4% in those with three, and 52.9%–85.7% in those with four failing organ systems, depending on which organ systems were failing. The variation in mortality according to the different sets of OFs decreased towards 12‐month mortality observation.

## 4 DISCUSSION

We evaluated Finnish ICU patients’ SOFA scores during the first 24 h in the ICU and assessed the prevalence of different OFs, defined as the organ system‐specific SOFA score of 3 or 4, and their associations with mortality. Cardiovascular failure, observed in 53% of patients, was the most common, followed by respiratory failure (23%), neurologic (18%), renal failure (8%), coagulation failure (3%) and hepatic failure (1%).

Mortality increased with increasing SOFA scores. However, scores reflecting dysfunctions of different organ systems were not equivalent as metrics of risk. In particular, high cardiovascular SOFA scores did not imply as high a risk of death as high scores of other SOFA components. In addition, OF combinations including cardiovascular failure were associated with lower mortality than other OF combinations: hospital mortality was in the range 25%–45% for patients with cardiovascular failure together with another OF, whereas mortality exceeded 50% for all other OF combinations except the combination of neurologic and respiratory failure (40%). Moreover, within a group of patients with comparable total SOFA scores, the risk of death was lower in patients with cardiovascular OF compared with patients with other OFs in patients with a total SOFA score lower than 12.

The contributions of SOFA component scores to outcome has not been studied much previously. However, our findings contradict those of the 1999 study by the Working Group on Sepsis Related Problems, where cardiovascular SOFA scores contributed more strongly than scores of other components to poor outcomes.^3^ On the contrary, our results are in accordance with the study by Gupta et al. on 2796 septic patients with in‐hospital mortality of 10%. Coagulation dysfunction or failure predicted a higher and cardiovascular dysfunction or failure a lower risk increase compared with dysfunctions of other organ systems.^22^

Recently, Bachmann et al. found that there are few patients with 2 cardiovascular SOFA points, and the prognostic value of cardiovascular SOFA was poor in patients assessed for intra‐abdominal hypertension and gastrointestinal dysfunction.^23^, ^24^, ^25^ Our findings in a large sample of general ICU patients confirm this. The distribution of the cardiovascular SOFA score had two peaks, made up of categories 0−1 and 3−4. A score of 2 was uncommon, present for roughly 1% of the patients. Two cardiovascular SOFA points are scored to patients who are administered dopamine at a dose less than 5µg kg^−1^ · min^−1^ or dobutamine at any dose. Recent guidelines recommend against or advise specific caution for monotherapy use of these inotropes in circulatory shock.^26^, ^27^ However, administering dopamine to brain‐dead organ donors with the intention to support renal function was relatively common in Finland during the study period,^28^ which may partly explain the high mortality in this SOFA category.

Outcomes of ICU patients have improved over the years. In 1998, Vincent et al. reported an ICU mortality of 90% in patients with a SOFA score above 15,^2^ whereas in‐hospital mortality for patients with first‐day SOFA score above 15 was 72% in our study. The overall ICU and in‐hospital mortality was lower in our study compared with that reported in the 1990s.^2^, ^3^ In addition to assumed improvements in prognosis of ICU patients, a plausible explanation for this is that we also included patients with preceding scheduled surgery. Our results show, however, that the cardiovascular SOFA score was associated with lower risk of mortality in the whole cohort, in both emergency and elective admissions, as well as those with at least 48 h length of ICU stay.

Although high SOFA scores often indicate a poor prognosis, cardiovascular scores seem to be an exception. This may reflect a change in clinical practices in recent years. The SOFA score was introduced in an era of more restricted use of vasopressors. During the last two decades, the use of norepinephrine has become more common.^29^, ^30^, ^31^ Vasopressor treatment is initiated earlier without preceding large doses of resuscitation fluids.^32^, ^33^, ^34^, ^35^ An infusion of norepinephrine lasting at least one hour, even at a small dose, assigns 3 points to the cardiovascular component of the SOFA score. Moreover, an infusion rate exceeding 0.1 µg kg^−1^·min^−1^, which is not a particularly high dose in contemporary intensive care, gives four points. Because of this change in clinical practice, the cardiovascular SOFA score seems to have suffered from inflation. This could also explain the divergence of our findings from those made by Moreno et al.^3^ more than two decades ago.

Risk of death increases with an increasing amount of failing organ systems.^36^, ^37^ Our findings imply that some OFs are more likely to occur concurrently than other failures. Moreover, mortality was dependent on which organ systems were failing. The Working group on sepsis‐related problems demonstrated a pattern for concurrently occurring OFs by means of principal components analysis.^3^ The group identified two common OF combinations. The first combination comprised respiratory, cardiovascular and neurologic OFs, whereas the second comprised coagulation, hepatic and renal OFs. In our study, this first combination of respiratory, cardiovascular and neurologic OFs was also the most common of the combinations with three OFs, affecting 37% of patients with at least three concurrently failing organ systems. The in‐hospital and 12‐month mortalities associated with this particular combination were 41% and 55%, respectively, whereas in‐hospital and 12‐month mortalities of patients with other triple OF combinations ranged between 56%–82% and 63%–88%, respectively.

We found that the second combination, which comprised coagulation, hepatic and renal OFs, occurred 44 times more often than one would have expected by observing merely the frequency of these OFs in the whole study population.

There is growing interest in using the SOFA score as a surrogate endpoint for mortality in clinical trials.^17^ Our findings suggest that this may not be without problems. Regarding risk of death, weights of different SOFA component scores are different, and the prognosis of patients with multi‐OF is dependent on which organ systems fail. In particular, cardiovascular SOFA scores do not signal cardiovascular dysfunction of equivalent severity to dysfunctions reflected by similar scores of other organ systems. The scoring criteria of cardiovascular dysfunction/failure may need an update. However, we must be aware that changing even one of the SOFA components would practically create a second version of the SOFA score. This might improve the measurement of organ dysfunctions but also mean that we would lose comparability to previous studies that have used the original SOFA score.

### 4.1 Strengths and limitations of the study

Our study population consisted of a large unselected group of patients treated in Finnish ICUs. The data were retrieved from a high‐quality national database with all Finnish general ICUs participating. Therefore, our study population is well representative of adult ICU patients in Finland. We do not know whether the results are generalizable to other countries. However, early use of norepinephrine has become more common in other countries as well,^31^ and it is likely that the relation between cardiovascular SOFA scores and mortality may have weakened also in other countries.

A major limitation of our study is that the SOFA scores were based only on measurements during the first 24 h after admission to the ICU, whereas previous studies have shown that a change in SOFA score over time is the most reliable predictor of mortality.^38^, ^39^

## 5 CONCLUSION

All SOFA components are associated with mortality. However, high cardiovascular SOFA scores did not mean as high a risk of death as high scores of other SOFA components. Moreover, OF combinations including cardiovascular failure were associated with lower mortality than other OF combinations. OFs are likely to occur concurrently. The scoring of cardiovascular dysfunction needs to be updated.

## CONFLICTS OF INTEREST

None.

## AUTHOR CONTRIBUTIONS

MR presented the first idea of the study. AP analysed and interpreted the data, supported by MR and PP. AP drafted the first version of the manuscript and created the figures. TS contributed in statistical analysis. JT helped in interpreting the results. All authors revised the manuscript and read and approved the final manuscript.

## Supporting information

Supplementary Material
