# Supplementary material for: Association of Sequential Organ Failure Assessment (SOFA) components with mortality
Source: Acta Anaesthesiol Scand. 2022 Apr 10;66(6):731–41. doi: 10.1111/aas.14067 (PMC9322581; doi:10.1111/aas.14067)
Supplement: Supplementary file 1 — Supplementary Material [file AAS-66-731-s001.docx]

**Supplementary materials**

**Association of Sequential Organ Failure Assessment (SOFA) components with mortality**

**Authors**

Anssi Pölkki¹^,2^; Pirkka T. Pekkarinen^3^; Jukka Takala^4^; Tuomas Selander^5^; Matti Reinikainen^1,2^

**Institutions**

^1^Department of Anaesthesiology and Intensive Care, Kuopio University Hospital, Kuopio, Finland; ^2^ University of Eastern Finland, Kuopio, Finland; ^3^Division of Intensive Care Medicine, Department of Anaesthesiology, Intensive Care and Pain Medicine, University of Helsinki and Helsinki University Hospital, Helsinki, Finland, ^4^ Department of Intensive Care Medicine, University Hospital Bern (Inselspital), University of Bern, Bern, Switzerland; Science Service Center, Kuopio University Hospital, Kuopio, Finland

**Table of Contents**

| **Supplemental Table 1** | The description of Sequential Organ Failure Score (SOFA) | 2 |
| --- | --- | --- |
| **Supplemental Table 2. Panel 1.** | Data on patients with at least 2 concurrent organ failures | 3 |
| **Supplemental Table 2. Panel 2.** | Data on patients with at least 3 concurrent organ failures | 4 |
| **Supplemental Table 2. Panel 3.** | Data on patients with at least 4 concurrent organ failures | 5 |
| **Supplemental Table 3.** | Occurrence of organ failures and the corresponding hospital mortalities in different admission categories | 6 |

**Supplemental Table 1.** The description Sequential Organ Failure Assessment (SOFA). The score was introduced by Vincent et al. [1] in 1996.

| **SOFA score** | **1** | **2** | **3** | **4** |  |
| --- | --- | --- | --- | --- | --- |
| *Respiration* |  |  |  |  |  |
| PaO_2_/FiO_2_, mmHg | <400 | <300 | <200* | <100* |  |
| *Coagulation* |  |  |  |  |  |
| Platelets ×10^3^/mm^3^ | <150 | <100 | <50 | <20 |  |
| *Hepatic* |  |  |  |  |  |
| Bilirubin, mg/dL | 1.2-1.9 | 2.0-5.9 | 6.0-11.9 | >12.0 |  |
| (µmol/l) | 20-32 | 33-101 | 102-204 | >204 |  |
| *Cardiovascular* |  |  |  |  |  |
| Hypotension | MAP <70 | Dopamine ≤5 or dobutamine (any)** | Dopamine >5 or norepinephrine ≤0.1 or epinephrine ≤0.1 ** | Dopamine >15 or norepinephrine >0.1 or epinephrine >0.1 ** |  |
| *Neurologic* |  |  |  |  |  |
| Glasgow Coma Score | 13-14 | 10-12 | 6-9 | <6 |  |
| *Renal* |  |  |  |  |  |
| Creatinine, mg/dL (mmol /l) or urine output | 1.2-1.9  (110-170) | 2.0-3.4  (171-299) | 3.5-4.9 (300-440) or  <500 ml/day | >5.0 (>440) or  <200 ml/day |  |

**Patients treated with mechanical respiratory support*

***Adrenergic agents administered for at least 1 hr (doses given in μg/kg/min).*

Abbreviations: PaO_2_, Partial arterial oxygen saturation; MAP, Mean arterial pressure.

**Supplemental Table S2.** Patients with at least two (Panel 1), at least three (Panel 2) and at least four (Panel 3) concurrent organ failures (OF). The columns present the numbers of observed (Obs.) patients with the particular OFs; the numbers of patients expected (Exp.) to have that particular OF combination, assuming that the OFs appear independently of each other; the observed/expected ratio, i.e. the Standardized Occurrence Ratio (SOR); its 95% confidence interval CI); and the Bonferroni corrected p-values. The rightmost columns present the in-hospital and 12-month mortalities.

**Supplemental Table 2. Panel 1.** Data on patients with at least 2 concurrent organ failures

|  | Obs. | Exp. | SOR | 95% CI | p | In-hospital Mortality | 12-month Mortality |
| --- | --- | --- | --- | --- | --- | --- | --- |
| Respiratory & Coagulation | 611 | 413.9 | 1.48 | 1.36-1.60 | <0.001 | 318/611 (52%) | 366/582 (62.9%) |
| Respiratory & Cardiovascular | 11293 | 7952.3 | 1.42 | 1.39-1.45 | <0.001 | 2907/11293 (25.7%) | 3956/10773 (36.7%) |
| Respiratory & Neurologic | 4201 | 2737.6 | 1.53 | 1.49-1.58 | <0.001 | 1666/4201 (39.7%) | 2160/3985 (54.2%) |
| Respiratory & Renal | 1868 | 1220.8 | 1.53 | 1.46-1.69 | <0.001 | 1013/1868 (54.2%) | 1141/1773 (64.4%) |
| Respiratory & Hepatic | 161 | 139.7 | 1.15 | 0.98-1.34 | 1.000 | 89/161 (55.3%) | 108/158 (68.4%) |
| Coagulation & Cardiovascular | 1220 | 984.6 | 1.24 | 1.17-1.31 | <0.001 | 521/1220 (42.7%) | 612/1151 (53.2%) |
| Coagulation & Neurologic | 351 | 338.9 | 1.04 | 0.93-1.15 | 1.000 | 202/351 (57.5%) | 235/328 (71.6%) |
| Coagulation & Renal | 465 | 151.1 | 3.08 | 2.80-3.37 | <0.001 | 268/465 (57.6%) | 293/444 (66%) |
| Coagulation & Hepatic | 142 | 17.3 | 8.21 | 6.91-9.68 | 0.001 | 81/142 (57%) | 91/135 (67.4%) |
| Cardiovascular & Neurologic | 7477 | 6512.7 | 1.15 | 1.12-1.17 | <0.001 | 2451/7477 (32.8%) | 3425/7101 (48.2%) |
| Cardiovascular & Renal | 3614 | 2904.1 | 1.24 | 1.20-1.29 | <0.001 | 1641/3614 (45.4%) | 2006/3442 (58.3%) |
| Cardiovascular & Hepatic | 436 | 332.4 | 1.31 | 1.19-1.44 | <0.001 | 198/436 (45.4%) | 261/419 (62.3%) |
| Neurologic & Renal | 1114 | 999.8 | 1.11 | 1.05-1.18 | 0.017 | 726/1114 (65.2%) | 799/1046 (76.4%) |
| Neurologic & Renal | 122 | 114.4 | 1.07 | 0.89-1.27 | 1.000 | 72/122 (59%) | 91/119 (76.5%) |
| Renal & Hepatic | 231 | 51.0 | 4.53 | 3.96-5.15 | <0.001 | 128/231 (55.4%) | 155/222 (69.8%) |

**Supplemental Table 2. Panel 2.** Data on patients with at least 3 concurrent organ failures

|  | Obs. | Exp. | SOR | 95% CI | p | In-hospital Mortality | 12-month Mortality |
| --- | --- | --- | --- | --- | --- | --- | --- |
| Respiratory & Coagulation & Cardiovascular | 542 | 219.8 | 2.47 | 2.26-2.86 | <0.001 | 291/542 (53.7%) | 326/516 (63.2%) |
| Respiratory & Coagulation & Neurologic | 160 | 75.7 | 2.11 | 1.80-2.47 | <0.001 | 103/160 (64.4%) | 119/154 (77.3%) |
| Respiratory & Coagulation & Renal | 228 | 33.7 | 6.76 | 5.91-7.70 | <0.001 | 155/228 (68%) | 164/218 (75.2%) |
| Respiratory & Coagulation & Hepatic | 43 | 3.9 | 11.14 | 8.06-15.00 | <0.001 | 30/43 (69.8%) | 33/42 (78.6%) |
| Respiratory & Cardiovascular & Neurologic | 3265 | 1453.7 | 2.25 | 2.17-2.32 | <0.001 | 1353/3265 (41.4%) | 1722/3113 (55.3%) |
| Respiratory & Cardiovascular & Renal | 1616 | 648.2 | 2.49 | 2.37-2.62 | <0.001 | 931/1616 (57.6%) | 1029/1539 (66.9%) |
| Respiratory & Cardiovascular & Hepatic | 140 | 74.2 | 1.89 | 1.59-2.23 | <0.001 | 82/140 (58.6%) | 100/137 (73%) |
| Respiratory & Neurologic & Renal | 594 | 223.2 | 2.66 | 2.45-2.88 | <0.001 | 442/594 (74.4%) | 461/560 (82.3%) |
| Respiratory & Neurologic & Hepatic | 53 | 25.5 | 2.07 | 1.55-2.71 | <0.001 | 31/53 (58.5%) | 37/53 (69.8%) |
| Respiratory & Renal & Hepatic | 79 | 11.4 | 6.94 | 5.49-8.64 | <0.001 | 53/79 (67.1%) | 58/77 (75.3%) |
| Coagulation & Cardiovascular & Neurologic | 279 | 180.0 | 1.55 | 1.37-1.74 | <0.001 | 174/279 (62.4%) | 190/261 (72.8%) |
| Coagulation & Cardiovascular & Renal | 385 | 80.3 | 4.80 | 4.33-5.30 | <0.001 | 244/385 (63.4%) | 261/371 (70.4%) |
| Coagulation & Cardiovascular & Hepatic | 108 | 9.2 | 11.76 | 9.64-14.19 | <0.001 | 67/108 (62%) | 73/105 (69.5%) |
| Coagulation & Neurologic & Renal | 116 | 27.6 | 4.20 | 3.47-5.04 | <0.001 | 92/116 (79.3%) | 96/112 (85.7%) |
| Coagulation & Neurologic & Hepatic | 35 | 3.2 | 11.07 | 7.71-15.39 | <0.001 | 24/35 (68.6%) | 28/34 (82.4%) |
| Coagulation & Renal & Hepatic | 62 | 1.4 | 43.97 | 33.71-56.36 | <0.001 | 41/62 (66.1%) | 43/59 (72.9%) |
| Cardiovascular & Neurologic & Renal | 882 | 530.9 | 1.66 | 1.55-1.77 | <0.001 | 605/882 (68.6%) | 657/831 (79.1%) |
| Cardiovascular & Neurologic & Hepatic | 93 | 60.8 | 1.53 | 1.24-1.87 | 0.006 | 56/93 (60.2%) | 73/91 (80.2%) |
| Cardiovascular & Renal & Hepatic | 194 | 27.1 | 7.16 | 6.19-8.24 | <0.001 | 109/194 (56.2%) | 136/189 (72%) |
| Neurologic & Renal & Hepatic | 51 | 9.3 | 5.47 | 4.07-7.19 | <0.001 | 42/51 (82.4%) | 44/50 (88%) |

**Supplemental Table 2. Panel 3.** Data on patients with at least 4 concurrent organ failures

|  | Obs. | Exp. | SOR | 95% CI | p | In-hospital Mortality | 12-month Mortaliy |
| --- | --- | --- | --- | --- | --- | --- | --- |
| Respiratory & Coagulation & Cardiovascular & Neurologic | 141 | 40.2 | 3.51 | 2.95-4.14 | <0,001 | 94/141 (66.7%) | 105/135 (77.8%) |
| Respiratory & Coagulation & Cardiovascular & Renal | 217 | 17.9 | 12.11 | 10.56-13.84 | <0,001 | 147/217 (67.7%) | 156/207 (75.4%) |
| Respiratory & Coagulation & Cardiovascular & Hepatic | 41 | 2.1 | 20.00 | 14.35-27.13 | <0,001 | 29/41 (70.7%) | 31/40 (77.5%) |
| Respiratory & Coagulation & Neurologic & Renal | 70 | 6.2 | 11.35 | 8.85-14.34 | <0,001 | 55/70 (78.6%) | 59/66 (89.4%) |
| Respiratory & Coagulation & Neurologic & Hepatic | 17 | 0.7 | 24.08 | 14.03-38.56 | <0,001 | 9/17 (52.9%) | 12/17 (70.6%) |
| Respiratory & Coagulation & Renal & Hepatic | 26 | 0.3 | 82.61 | 53.96-121.04 | <0,001 | 20/26 (76.9%) | 21/25 (84%) |
| Respiratory & Cardiovascular & Neurologic & Renal | 535 | 118.5 | 4.51 | 4.14-4.91 | <0,001 | 400/535 (74.8%) | 415/504 (82.3%) |
| Respiratory & Cardiovascular & Neurologic & Hepatic | 42 | 13.6 | 3.10 | 2.23-4.19 | <0,001 | 26/42 (61.9%) | 32/42 (76.2%) |
| Respiratory & Cardiovascular & Renal & Hepatic | 74 | 6.1 | 12.24 | 9.61-15.36 | <0,001 | 49/74 (66.2%) | 55/72 (76.4%) |
| Respiratory & Neurologic & Renal & Hepatic | 24 | 2.1 | 11.53 | 7.39-17.15 | <0,001 | 20/24 (83.3%) | 20/24 (83.3%) |
| Coagulation & Cardiovascular & Neurologic & Renal | 106 | 14.7 | 7.23 | 5.92-8.74 | <0,001 | 85/106 (80.2%) | 89/102 (87.3%) |
| Coagulation & Cardiovascular & Neurologic & Hepatic | 30 | 1.7 | 17.87 | 12.05-25-50 | <0,001 | 21/30 (70%) | 24/29 (82.8%) |
| Coagulation & Cardiovascular & Renal & Hepatic | 56 | 0.8 | 74.79 | 56.49-97.12 | <0,001 | 38/56 (67.9%) | 41/55 (74.5%) |
| Coagulation & Neurologic & Renal & Hepatic | 14 | 0.3 | 54.31 | 29.69-91.13 | <0,001 | 12/14 (85.7%) | 13/14 (92.9%) |
| Cardiovascular & Neurologic & Renal & Hepatic | 39 | 5.0 | 7.87 | 5.60-10.76 | <0,001 | 32/39 (82.1%) | 36/39 (92.3%) |

**Supplemental Table 3. Occurrence of organ failures and the corresponding hospital mortalities in different admission categories**

|  | **Respi-ratory** | **Coagu-lation** | **Hepatic** | **Cardio-vascular** | **Neuro-logic** | **Renal** |
| --- | --- | --- | --- | --- | --- | --- |
| **Medical** | 8,879 (22.3%) | 1,363 (3.8%) | 506 (1.4%) | 16,044 (43.0%) | 10,095 (27.6%) | 4,240  (11.4%) |
| Mortality | 29.7% | 38.1% | 44.5% | 25.6% | 29.3% | 36.4% |
| **Elective surgical** | 2,665 (15.2%) | 117 (0.7%) | 16 (0.1%) | 11,542 (65.7%) | 311 (1.8%) | 257 (1.5%) |
| Mortality | 4.9% | 12.8% | 12.5% | 2.2% | 8.7% | 19.8% |
| **Emergency surgical** | 2,916 (24.4%) | 314  (2.7%) | 103 (0.9%) | 7,792 (65.1%) | 1,614 (13.9%) | 963 (8.0%) |
| Mortality | 20.6% | 31.2% | 23.3% | 15.3% | 24.2% | 38.7% |

The occurrence of different organ failures and the associated hospital mortalities are presented for each admission category. Overall in-hospital mortality was 15.2% in the medical group, 1.5% in the elective surgical group and 10.8% in the emergency surgical group.
